# Examining the Effects of Homochirality for Electron Transfer in Protein Assemblies

**DOI:** 10.1021/acs.jpcb.3c02913

**Published:** 2023-07-18

**Authors:** Jimeng Wei, Brian P. Bloom, Wiley A. Dunlap-Shohl, Caleb B. Clever, José E. Rivas, David H. Waldeck

**Affiliations:** Department of Chemistry, University of Pittsburgh, Pittsburgh, Pennsylvania 15260, United States

## Abstract

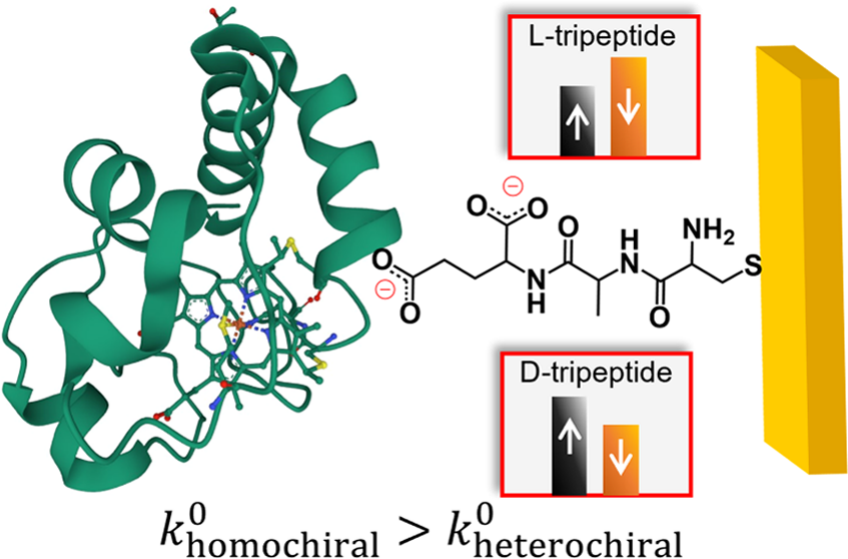

Protein voltammetry studies of cytochrome *c*, immobilized on chiral tripeptide monolayer films, reveal the importance of the electron spin and the film’s homochirality on electron transfer kinetics. Magnetic film electrodes are used to examine how an asymmetry in the standard heterogeneous electron transfer rate constant arises from changes in the electron spin direction and the enantiomer composition of the tripeptide monolayer; rate constant asymmetries as large as 60% are observed. These findings are rationalized in terms of the chiral induced spin selectivity effect and spin-dependent changes in electronic coupling. Lastly, marked differences in the average rate constant are shown between homochiral ensembles, in which the peptide and protein possess the same enantiomeric form, compared to heterochiral ensembles, where the handedness of the peptide layer is opposite to that of the protein or itself comprises heterochiral building blocks. These data demonstrate a compelling rationale for why nature is homochiral; namely, spin alignment in homochiral systems enables more efficient energy transduction.

## Introduction

Redox reactions are ubiquitous in nature and play an essential role in biochemical processes, including bioenergetics and photosynthesis.^1^ Proteins immobilized on the surface of working electrodes coated with self-assembled monolayers (SAMs) are widely used to mimic fundamental features of electron transfer in biological systems.^2−5^ For compact and insulating SAMs, the electron transfer proceeds by electron tunneling through the SAM,^3,6,7^ and the standard heterogeneous electrochemical rate constant can be described using Marcus theory.^3,4^ Although electron transfer reactions in biology are well known, their possible connection with homochirality in biomolecules and their assemblies is not. It is established that natural biological assemblies are predominantly composed of l-amino acid and peptide building blocks, but why nature expresses this preference for homochirality is still a matter of debate. The discovery of chiral induced spin selectivity (CISS),^8^ and its manifestation in biomolecules,^9,10^ motivates the examination of the connection between homochirality and long-range electron transport in supramolecular assemblies of chiral biomolecules. We hypothesize that chirality-based spin-filtering can affect electron transfer rates of biomolecular assemblies, somewhat like a spin valve does in a conventional circuit. That is, in heterochiral assemblies, the electron spin orientations conducive to transport in a certain direction abruptly shift (as in a spin valve with electrodes of opposite magnetization), reducing the overall electron transfer rate. By contrast, homochiral systems maintain a consistent favorable spin orientation across the entire assembly (as in a spin valve with electrodes of the same magnetization), thereby enhancing electron transfer. This work studies the electron transfer through monolayer-coated electrodes to the immobilized protein cytochrome *c*, as a model system for biological interfaces in which the enantiomeric form of the monolayer film’s constituent molecules can be modified.

The CISS effect implies a connection between the electron spin and the efficiency of electron transmission through chiral molecules and chiral supramolecular constructs.^9^ While CISS was not addressed in the study of electron transfer before the early 2000s, it has since been shown to manifest for electron transfer in DNA, oligopeptides, small chiral molecules, and chiral inorganic materials.^11−17^ More recently, CISS was demonstrated in biomacromolecular systems. Naaman and co-workers showed that the electron conduits in cytochrome proteins MtrF and OmcA, from the bacterium *Shewanella oneidensis* MR-1, are spin-polarized;^18^ and similar spin-mediated effects have been probed using electrochemical experiments on proteins, such as laccase, cytochrome *c*, and bacteriorhodopsin.^19^ Spin constraints arising from the CISS effect can also affect electron transfer kinetics. For instance, Bloom et al. showed how the photoinduced electron transfer rate to a chiral nanoparticle acceptor moiety, in a donor–bridge–acceptor assembly, depends on the sense of the circularly polarized light (clockwise *vs* counterclockwise) used to excite the donor nanoparticle.^20^ A similar phenomenon was shown in electrochemical experiments. Tassinari et al. reported a chirality-dependent asymmetry in the electron transfer rate between ferrocene and a gold substrate tethered through an oligopeptide SAM.^21^ While these experiments demonstrate that the chiral components in biomolecules and biomolecular systems possess spin effects, a relationship between the homochirality of such assemblies and CISS has not been reported. This work uses a modular approach to probe the role of spin polarization during electron transfer between cytochrome *c* (which inherently possesses levorotatory chirality) and ferromagnetic electrodes via short oligopeptide monolayers. Spin constraints on the electron transfer are revealed by magnetizing the electrode parallel or antiparallel to the direction of the electron current, and the effect of the film’s chirality on the electron transfer is explored by changing the enantiomeric form of the amino acids composing the oligopeptide molecules in the monolayer.

Globular cytochrome *c* (Cyt *c*), with its heme iron, was chosen as the redox couple because of its well-characterized structure and electron transfer kinetics.^22,23^ The immobilization of Cyt *c* onto a SAM can be achieved in several ways, including electrostatic immobilization between the Cyt *c*’s surface lysine residues and the carboxylate termini of a SAM,^24^ amide bond formation between the Cyt *c* surface and the SAM,^25^ and ligation between the Cyt *c*’s heme iron and a nitrogen ligand of the SAM.^26^ Electrostatic assembly is believed to most closely resemble Cyt *c*’s function *in vivo* as an electron transport protein in the inner membrane of mitochondria^23,27^ and hence was the method chosen for this work. Recent work by Clark and co-workers showed that the electrochemical rate constants for Cyt *c* immobilized on the surface of SAMs comprising Cys-Ala-Glu tripeptides are significantly different from that of 11-mercaptoundecanoic acid (11-MUA) SAMs, despite their overall length being similar.^22,28^ Motivated by these studies, this work explores the electron transfer between Cyt *c* and a ferromagnetic electrode, across tripeptide SAMs, as a function of the electrode magnetization and the enantiomeric form (levorotatory, L, *vs* dextrorotatory, D) of the individual amino acids forming the tripeptide. Changes in the electron transfer kinetics with electrode magnetization are observed and indicate that the charge transport across the tripeptide SAM–Cyt *c* assembly is spin-polarized. The spin effects present in the peptide assemblies are used to rationalize the difference in kinetics between homochiral systems with those possessing varying degrees of heterochirality. In particular, the average charge transfer rate is much faster for homochiral (LLL-tripeptide and L-protein) assemblies, compared to heterochiral (DDD-tripeptide and L-protein or LDL-tripeptide and L-protein) assemblies, for which the average rate constant is reduced by nearly an order of magnitude. These results demonstrate that homochiral assemblies confer a major advantage in facilitating electron transfer, providing a plausible explanation for their prevalence in nature.

## Experimental Section

### Hall Effect Device Fabrication and Measurement

Hall effect devices were fabricated as reported previously^29^ and cleaned by boiling in acetone (99.5%, Fisher) and in 2-propanol (99.5%, Fisher Chemical), rinsed in 2-propanol and water, and dried under a stream of Ar gas (90–99%, Matheson Tri-Gas, Inc.). The devices were then oxidized in a UV/Ozone cleaner (UV. TC. NA. 003, Bioforce Nanoscience Inc.) for 2 min and placed in ethanol for at least 30 min prior to incubation. The device was incubated in a 3.5 mM oligopeptide (95%, Genemed Synthesis Inc.) solution in ethanol (200 proof, Fisher Chemical) for 48 h. Following incubation, the coated Au surface was rinsed with 3 M KCl (99%, Fisher Chemical), followed by deionized H_2_ O, and then dried under an Ar stream. A polydimethylsiloxane (PDMS) (Sylgard 184) electrochemical cell was assembled around the device and cured for 18 h at 45 °C.

Measurements were conducted in 100 mM tetrabutylammonium hexafluorophosphate (TBAPF_6_) (98%, Sigma-Aldrich) electrolyte in acetonitrile (99.8%, anhydrous, Sigma-Aldrich). Using a Keithley 2636 source measure unit, a constant current of 50 μA is applied between the “source” and “drain”, while a polarizing “gate” voltage is applied perpendicular to both the source–drain current and the Hall voltage probes. The voltage was electrically insulated from the solution by a ∼0.18 mm thick glass slide. The Hall voltage is measured using a Keithley Nanovoltmeter 2182A device. The direction of the source–drain current was then reversed, and the measurements repeated, to account for any asymmetry in the device.

### Electrode Fabrication, SAM Preparation, and Cytochrome *c* Assembly

The working electrodes were fabricated by evaporating 100 nm of Ni, followed by 5 nm of Au, onto a glass microscope slide (Fisher Scientific) or silicon wafer (University Wafer Inc.) that possessed a 5 nm Ti adhesion layer using a Plassys electron beam evaporator MEB550S. Following evaporation, the electrode was fixed onto the bottom of an electrochemical cell using silicone caulk (General Electric) and allowed to cure overnight. The circular cutout on the bottom of the cell has a diameter of ∼6 mm and acts to define the geometric active area (∼0.28 cm^2^) for the working electrode. After the silicone caulk was cured, a SAM solution containing either pure oligopeptide or oligopeptide mixed with a diluent was added to the electrochemical cell and incubated for 48 h. The incubation solution for the assembly of the pure SAM was 2 mM of oligopeptide dissolved in ethanol. For mixed SAMs, the concentration of the oligopeptide was the same, but the solution also included a diluent molecule, 1.5 mM 6-mercapto-1-hexanol (98%, Tokyo Chemical Industry). Following incubation, the electrode was rinsed with fresh ethanol and 4.4 mM phosphate buffer solutions (pH = 7, 99.8%, Fisher Chemical). To immobilize Cyt *c* (95%, Sigma-Aldrich) on top of the SAM, a solution comprising 30 μM equine heart Cyt *c* in 4.4 mM phosphate buffer was added to the washed electrochemical cell and incubated for at least 1 h. The cell was then rinsed with 40 mM KCl solution to remove any weakly adsorbed Cyt *c*.

### SAM and Tripeptide Characterization

X-ray photoelectron spectroscopy measurements were performed using a Thermo Fisher ESCALAB 250 Xi instrument on Au electrodes and referenced to adventitious carbon (284.8 eV). The SAM surface coverage was determined using previously established methods.^30^ Circular dichroism (CD) spectroscopy was used to measure the chiroptical properties of the tripeptides using an Olis DSM 17 CD spectropolarimeter.

### Cyclic Voltammetry

All of the electrochemical measurements were performed using a CH Instruments 618b potentiostat in a CH Instruments 200B Faraday cage. The reference electrode was Ag/AgCl in 3 M KCl and the counter electrode was a Pt wire. The distance between the working electrode and the reference electrode was fixed at 4 mm. Prior to the cyclic voltammetry, a magnet with a strength of 0.5 T was placed directly beneath the working electrode and the magnet was oriented with either its north or south pole perpendicular to the electrode surface. The magnet was located outside the electrochemical cell, and changing the field’s orientation did not alter the cell arrangement or geometry. The cyclic voltammograms were collected over a potential range from −0.3 to 0.3 V, and the scan rate was varied from 40 to 6000 mV/s.

Furthermore, the accuracy of *k*^0^ depends sensitively on the peak potential and possible distortion of the peak assignment from non-Faradaic background current in each voltammogram. To reliably assign the peak potentials in the voltammogram, the analysis uses a background and Voigt peak fitting function; see the Supporting Information for a more detailed discussion and corresponding Python script used for analysis. Three separate scans at each scan rate for each electrode were averaged to characterize and mitigate uncertainty in the peak potential and peak currents. In addition, a minimum of three independent electrode preparations were made for each series of experiments. The *k*^0^ values are obtained by quantifying how the peak redox potential shifts with voltage scan rate at the working electrode and fitting this dependence by the Marcus theory prediction.^3,4^ This work examines how the *k*^0^ values change with the electrode’s magnetization and the enantiomeric form of the amino acids composing the tripeptide SAMs.

## Results and Discussion

**Figure 1:**
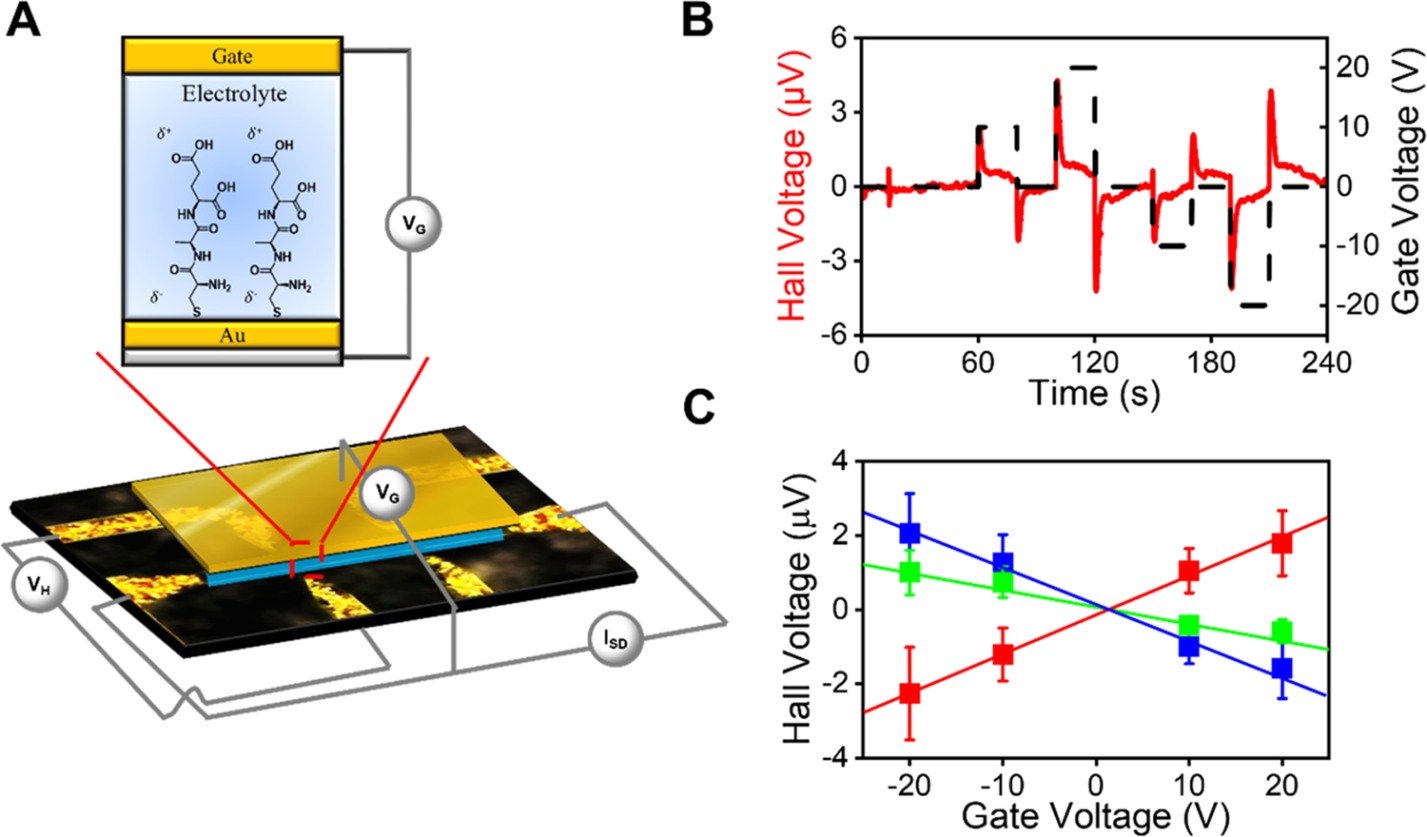
Overview of the Hall effect measurements. (A) Schematic view of the experimental setup for the Hall measurements. (B) Representative time profile of a measurement in which the square wave feature (black) shows the applied gate voltage *vs* time and the red waveforms show the corresponding Hall voltage response. (C) Plot of the measured Hall response for the initial spike after application of the gate voltage for LLL-(blue), DDD-(red), and LDL-tripeptide (green) SAMs. The error bars represent one standard deviation of the error over all measurements and devices at the applied gate voltage.

To demonstrate that the tripeptides used in this study exhibit an enantiospecific spin-filtering response (*i.e.*, the CISS effect), Hall measurements were performed. Figure 1A illustrates the experimental arrangement for our studies in which a monolayer of the tripeptide (Cys-Ala-Glu) is chemisorbed on an ultrathin (5 nm) Au film that coats a GaN substrate with an imbedded Hall bar circuit. The peptide-coated electrode is placed in an inert electrolyte solution and biased at a voltage with respect to a counter (or gate) electrode. Upon application of a bias voltage between the working electrode and the counter electrode, a charge displacement current flows in the SAM (double-layer charging current). If the charging current is spin-polarized, then it generates a magnetization on the working electrode’s surface and gives rise to a voltage between the Hall electrodes (the Hall voltage, *V*_H_), within the imbedded Hall circuit. For a layer of achiral molecules on the electrode surface, no magnetization (*V*_H_ is zero) is found; whereas a layer of chiral molecules on the surface gives rise to a nonzero *V*_H_ that is enantiospecific.^31^Figure 1B shows representative Hall voltage signals (red) that were measured for a DDD-tripeptide coated electrode, in which each amino acid is a d-enantiomer, on a working electrode at different applied gate voltages, in a 100 mM tetrabutylammonium hexafluorophosphate electrolyte in acetonitrile solution. The initial peak is chosen for analysis as the double-layer charging current, and hence the injected magnetization, is at its maximum.

Figure 1C shows the corresponding Hall voltage for LLL-tripeptide (blue) and DDD-tripeptide SAMs (red) under different gate voltages. Measurements were also performed on tripeptides with mixed handedness, L-Cys D-Ala L-Glu (LDL-tripeptide), and they are shown in green. Multiple measurements of each device as a function of the bias voltage and replicate measurements on multiple devices show that the Hall voltage changes systematically with the gate voltage; it displays a negative slope for LLL- and LDL-tripeptide SAMs and a positive slope for DDD-tripeptide SAMs. The LDL-tripeptide has a less negative slope than that of the LLL-tripeptide, which we attribute to its mixed chirality. The antisymmetry of the responses indicates that the LLL-tripeptide and LDL-tripeptide display electron density displacements with their spin aligned antiparallel to their velocity, whereas the DDD-tripeptide transmits electrons with their spin aligned parallel to the velocity.^32,33^ Because previous works have shown a correlation between the chiroptical properties of materials and their spin-filtering capabilities,^20^ and heterochiral tripeptides can adopt the handedness of either enantiomer,^34^ the circular dichroism of each tripeptide was measured (Figure S1). Here, the CD spectra of the LDL-tripeptide more closely resembles that of the LLL-tripeptide and is thus consistent with the spin polarization preference determined in the Hall device measurements.

**Figure 2:**
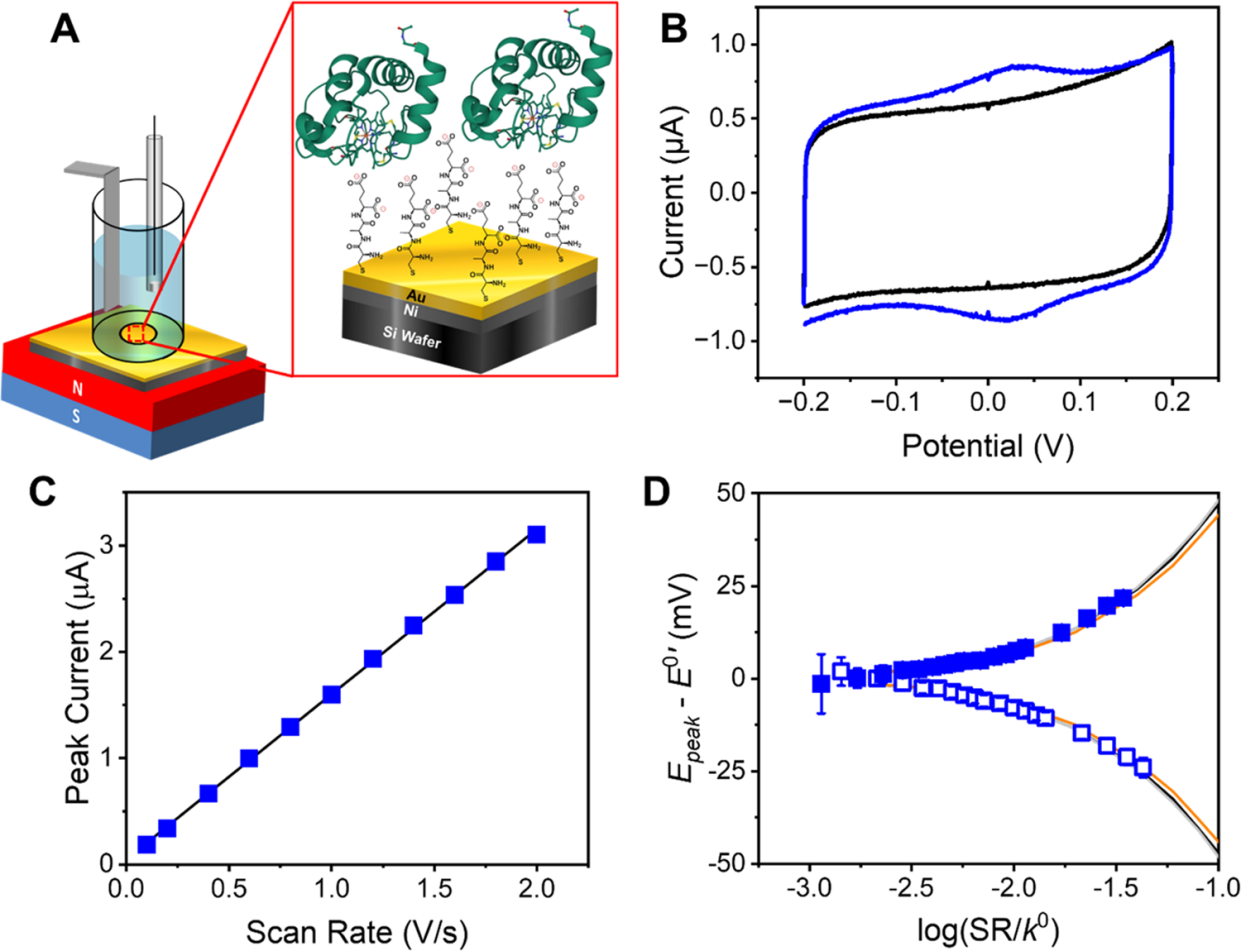
Panel A shows a diagram for the electrochemical setup and the SAM-coated working electrode. The tripeptide molecules were self-assembled on top of a Ni/Au film electrode, and the Cyt *c* was electrostatically immobilized on top of the SAM. The Cyt *c* structure shown is from the Protein Data Bank, code 3o20. (B) Cyclic voltammogram of an LLL-tripeptide SAM before (black) and after (blue) immobilization of Cyt *c* at 100 mV/s. (C) Plot of the peak current of Cyt *c* immobilized on an LLL-tripeptide SAM *vs* scan rate. The linear best fit line has an *R*^2^ value of 0.9995. (D) Plot of the Cyt *c* anodic (blue, solid squares) and cathodic (blue, open squares) peak potential shift as a function of the scan rate (SR) for an LLL-tripeptide Cyt *c* assembly. The error bars quantify uncertainty in the fitted voltammetric peak positions; see the Experimental Section for details. The solid curves are three different theoretical Marcus fits to the experimental data with reorganization energies of 0.1 eV (orange), 0.3 eV (black), and 0.6 eV (gray). The *R*^2^ values for the different reorganization energies are 0.90, 0.94, and 0.92, respectively.

Cyclic voltammetry was used to study the electron transfer kinetics of immobilized Cyt *c* through different tripeptide monolayers on a ferromagnetic working electrode. Figure 2A shows the experimental scheme in which a ferromagnetic electrode comprises a 100 nm Ni film and a 5 nm Au overlayer. The purpose of the Au overlayer is to protect the Ni from oxidation and to facilitate the assembly of the tripeptide SAM through cysteine-Au chemisorption; see the Supporting information for details regarding characterization of the tripeptide SAMs. In particular, X-ray photoelectron spectroscopy measurements demonstrate that the SAMs densely coat the electrode, and that the coverages for LLL- and DDD-tripeptide SAMs are equal to within the limits of instrumental error (Figure S2 and Table S1). The electrode is magnetized by applying an external magnetic field along the electrode’s surface normal, oriented either with the north or south pole toward the electrolyte. The magnetic field splits the spin sublevels of the working electrode’s electron distribution and makes the electrode interface sensitive to the spin state of an incoming electron. Oxidation (reduction) of the Cyt *c* heme unit proceeds by electron (hole) transfer from the immobilized Cyt *c* through the chiral SAM to the electrode.

Figure 2B shows cyclic voltammograms for LLL-tripeptide (black) and LLL-tripeptide/Cyt *c* assemblies (blue) using a 100 mV/s scan rate and pH 7 phosphate buffer supporting electrolyte, under a north magnetic field. The full width at half-maximum (FWHM) of 100.8 mV, after background subtraction, indicates that the Cyt *c* electron transfer is quasi-reversible. The Faradaic current has an integrated charge of 9.82 × 10^–8^ C and indicates a Cyt *c* coverage of 2.03 pmol/cm^2^. Figure S3 shows corresponding data for Cyt *c* immobilized on a mixed film of chiral LLL-tripeptide and achiral C6OH (6-mercapto-1-hexanol) diluent SAM and the case for Cyt *c* immobilized on an achiral control, 11-mercaptoundecanoic acid (11-MUA). Note that all of the data in Figure S3 were collected at 100 mV/s. The chiral SAM with a diluent has a Cyt *c* coverage of 2.01 pmol/cm^2^ and a FWHM of 115.8 mV, whereas the 11-MUA SAM has a Cyt *c* coverage of 0.84 pmol/cm^2^ and a FWHM of 112.1 mV. Note that reports of Cyt *c* redox properties on 11-MUA assemblies are comprehensive and consistent with previous results.^22,35−37^ A modest shift in the apparent redox potential is present in the different SAM compositions and is likely associated with differences in the charge density on the film.^38^

The standard electrochemical rate constant for Cyt *c* in these assemblies was obtained by measuring the shift in anodic and cathodic peak potential (*E*_p_) as a function of the scan rate. Figure 2C shows a plot of the peak current of the anodic wave of an LLL-tripeptide/Cyt *c* assembly *vs* the scan rate. The linear dependence between the peak current and the scan rate indicates that the Cyt *c* is immobilized on the monolayer surface, as opposed to free in solution. This experimental design simplifies the extraction of rate constants from the data by eliminating diffusion of the redox protein to the surface. Figure 2D shows a corresponding plot of the apparent anodic (blue, square) and cathodic (blue, open square) potential shift as a function of the scan rate. A fit to these data is then performed using Marcus theory, with the standard heterogeneous rate constant, *k*^0^, the formal potential, *E*^0^, and the reorganization energy, λ, as adjustable parameters.^23^ The data analysis, however, does not depend strongly on the reorganization energy; Figure 2D shows that a Marcus fit using λ = 0.1 eV (orange), 0.3 eV (black), and 0.6 eV (gray) does not give appreciable changes in the *R*^2^ values for the data fitting: 0.90, 0.94, and 0.92, respectively. For this reason, λ = 0.3 eV was used exclusively for the determination of *k*^0^, consistent with reported values in previous studies.^23,39^ With this choice, curve fitting is optimized by adjusting *k*^0^ and *E*^0^, which corresponds to the electron transfer rate constant and formal potential between the electrode and the iron cofactor of the protein at Δ*G* = 0.

**Figure 3:**
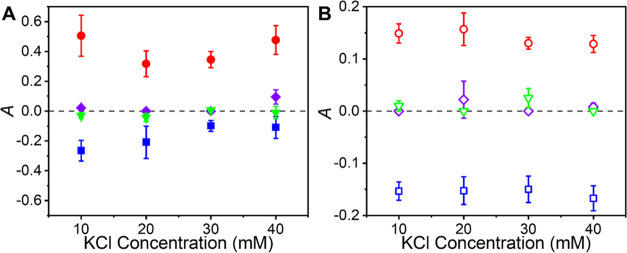
(A) Asymmetry in electron transfer rate constants is plotted *vs* the solution ionic strength for Cyt *c* immobilized on an LLL-tripeptide SAM (blue, square), DDD-tripeptide SAM (red, circle), LDL-tripeptide (green, triangle), and achiral 11-mercaptoundecanoic acid SAM (purple, diamond) at four different ionic strengths. (B) Asymmetry in electron transfer rates for Cyt *c* immobilized on mixed films of 6-mercaptohexanol with LLL-tripeptide SAM (blue, open square), DDD-tripeptide SAM (red, open circle), LDL-tripeptide (green, open triangle), and achiral 11-MUA SAM (purple, open diamond) at four different ionic strengths.

To probe the effect of spin on the electron transport, the ferromagnetic electrode was magnetized by an external magnet (0.5 T) such that its field was oriented normal to the electrode surface. The difference in rate constant with applied magnetic field orientation, north *vs* south, were compared through an asymmetry polarization parameter, *A*, defined as
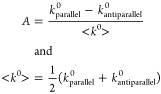
1where *k*_parallel_^0^ and *k*_antiparallel_^0^ correspond to rate constants in which the applied magnetic field causes the electron velocity to be aligned parallel or antiparallel to its spin, respectively. Here, placing the south pole of the magnet beneath the electrode corresponds to the parallel magnetization and north to the antiparallel magnetization. Figure 3A shows *A* determined for Cyt *c* immobilized on LLL-tripeptide SAMs (blue), DDD-tripeptide SAMs (red), LDL-tripeptide SAMs (green), and achiral 11-MUA SAMs (purple) collected at pH = 7 for four different solution ionic strengths. Tables S2 and S3 report the average *A* and number of trials for each ionic strength using pure and diluted SAMs, respectively. While the asymmetry parameter for a particular chiral SAM–Cyt *c* assembly shows variations with ionic strength, the asymmetry in electron transfer rate persists across different ionic strengths and indicates a magnetic field dependence, even as the solution resistance changes.

The data display a strong dependence of the asymmetry parameter on the composition of the SAM–Cyt *c* assembly components. For the LLL-tripeptide assemblies, *k*^0^ is larger when the electron transport is antiparallel to its spin, whereas the opposite is true for the DDD-tripeptide assemblies; *k*^0^ is larger when the electron transport is parallel to its spin. The change in sign of *A* associated with oligopeptide handedness demonstrates that the SAM-coated electrode acts as a source of spin-polarized electrons that interact in a spin-dependent manner with the Cyt *c*. Because variables like temperature and reorganization energy do not change significantly with the magnetic field direction, and Δ*G* = 0, the change in rate constants is assumed to arise from changes in the electronic coupling between the Cyt *c* and the SAM. In contrast, *k*^0^ values for 11-MUA and LDL-tripeptide assemblies are invariant with spin orientation. While the behavior of the LDL-tripeptide assemblies is surprising, considering the Hall response shown in Figure 1, the weaker spin polarization of the LDL oligopeptides may preclude rate constant asymmetries beyond the detection limit of our measurement system.

Figure 3B shows an analogous series of experiments to those in Figure 3A but instead uses mixed SAMs comprising C6OH diluent molecules and the tripeptides or 11-MUA. These data show the same general trend as the pure SAMs, where the achiral 11-MUA and the LDL-tripeptide containing assemblies show no significant rate asymmetry and the LLL-tripeptide and DDD-tripeptide containing assemblies show *A* of opposite signs. The magnitudes of the *A* for the LLL-tripeptides/C6OH and DDD-tripeptide/C6OH SAMs are lower than those found for the pure LLL-tripeptide and DDD-tripeptide SAMs; however, the magnitude of *A* for LLL-tripeptide/C6OH and DDD-tripeptide/C6OH are closer to one another than the pure tripeptide assemblies. Two distinct, but related, possible explanations for the reduced asymmetry are as follows:

- The voltammograms have contributions from current flow through both the achiral C6OH diluent and the tripeptides, and this feature dilutes the overall magnitude of *A*, *e.g.*, half of the current flows through the achiral C6OH (*A* ∼ 0) and the other half of the current flows through the homochiral tripeptide (|*A*| ∼ 0.30) and then the net asymmetry in rate constant would be polarized at |*A*| ∼ 0.15. Such an explanation requires that the immobilized protein is located near regions of the film with a significant percentage of C6OH. Given that the cross-sectional area of Cyt *c* is approximately 0.105 nm,^2,22^ it is plausible that it interacts with ∼13 SAM molecules in the film and some dilution of the tunneling current’s spin polarization is expected.
- Some theoretical models^40,41^ for spin-filtering of electrons through peptide SAMs on ferromagnetic electrodes posit that spin-filtering occurs at the FM electrode/chiral molecule boundary. In this case, the presence of achiral molecules in the film may reduce the net spin polarization of the electron current at the interface and hence the magnitude of the asymmetry in electrochemical rate constants.

Collectively, the data summarized in Figure 3 demonstrate that the electron current moving through the LLL-tripeptides and DDD-tripeptides is spin-polarized and that it depends on the magnetization of the electrode and the handedness of the chiral molecules, a hallmark of the CISS effect.

**Figure 4:**
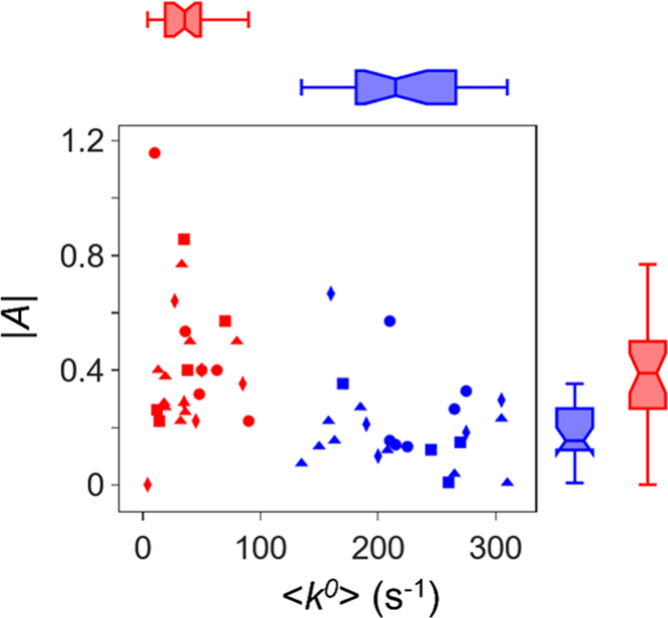
Plot of the magnitude of rate constant asymmetry, |*A*|, for LLL-tripeptide (blue) and DDD-tripeptide assemblies as a function of the average rate constant, <*k*^0^>, for different electrode preparations and solution conditions; 10 mM KCl (circles), 20mM KCl (diamonds), 30 mM KCl (triangles), and 40 mM KCl (squares). The horizontal blue and red box plots on top of the figure represent the statistics of <*k*^0^> values of LLL-tripeptide and DDD-tripeptide assemblies, respectively, and the vertical blue and red box plots on the right of the figure represent the statistics of |*A*| values of LLL-tripeptide and DDD-tripeptide assemblies, respectively. For each box plot, the central line represents the median of the data, the box represents the interquartile range (IQR), the whiskers extend to the extreme observed data points falling within 1.5 IQRs of the median, and the notches represent an estimate of the 95% confidence interval that can be used to characterize the statistical significance of differences among the populations.

In addition to a magnetic field dependence for the electrochemical rate constants in the LLL-tripeptide and DDD-tripeptide assemblies, we also observed a difference in the magnitudes of the average rate constants, *<k*^0^> (eq 1). While the electrochemical rate constants obtained for the tripeptides vary from one electrode preparation to the next, such behavior among differently prepared electrodes is not uncommon. For example, different works studying Cyt *c* immobilized on 11-MUA SAMs on Au substrates report *k*^0^ values that vary from 10 to 100 s^–1^.^22,35−37^ To explore whether the observed values of *k*^0^ affect the observed changes in *A*, extended trials for additional electrode preparations comprising LLL-tripeptide (blue) and DDD-tripeptide (red) assemblies were performed and are plotted in Figure 4 and in Tables S4 and S5. Each data point represents a single measurement, and variations in coverage and film structural quality among independently prepared electrodes are not minimized. Box and whisker plots are shown adjacent to the figure and illustrate that (i) the |*A*| does not possess a significant correlation with <*k*^0^>, (ii) the |*A*| is different for LLL-tripeptide and DDD-tripeptide assemblies, as neither the estimated 95% confidence intervals about the median nor the interquartile ranges overlap, and (iii) the <*k*^0^> values of LLL-tripeptide and DDD-tripeptide assemblies are significantly different. We posit that the difference in the |*A*| between LLL-tripeptide and DDD-tripeptide assemblies is associated with structural stereoisomeric effects, seeing as how the difference in asymmetry is minimized upon inclusion of a diluent (see Figure 3B). More strikingly, a large difference in <*k*^0^>, greater than 10-fold, is observed among the LLL-tripeptide and DDD-tripeptide assemblies (Table 1). The change in <*k*^0^> is reflected by the homochirality or heterochirality of the ensemble; namely, the handedness of the SAM and its constituents, with respect to the handedness of the protein. For homochiral assemblies (LLL-tripeptide/Cyt *c*), <*k*^0^> is fast; however, when the homochirality of the assembly is interrupted, either between the SAM and Cyt *c* or within the SAM itself, a reduction of rate constants occurs. These data imply that the spin-filtering effect of chiral building blocks in nature lead to more efficient electron transfer for homochiral systems.

**Table 1:** <*k*^0^> for Different SAMs, with 30 mM KCl

SAM composition	<*k*^0^>(s^–1^)
LLL only	209 ± 20
DDD only	33 ± 5
11-MUA only	31 ± 4
LDL only	22 ± 2

## Conclusions

This work establishes that assemblies comprising biological building blocks not only promote spin-filtering in electron transfer, but also increase the efficiency of electron transfer. Experiments on Cyt *c* assemblies immobilized on tripeptide SAMs in which all of the amino acids are the same enantiomer, *i.e.*, LLL-tripeptides and DDD-tripeptides, display a dependence of their electron transfer rate on the direction of a ferromagnetic electrode’s magnetization. Conversely, SAMs comprising molecules that are achiral or of mixed chirality, *e.g.*, 11-MUA or LDL-tripeptides, do not. Moreover, breaking the homochirality of the SAM–Cyt *c* assemblies, regardless of whether the interruption occurs between the tripeptide SAM and the Cyt *c* protein or within the tripeptide itself, causes a dramatic reduction in the electron transfer rate. Both the spin polarization and electron transfer rate effects arise from spin constraints during electron transmission, consistent with the chiral induced spin selectivity effect. This study shows that electron spin has profound importance for governing electron transfer processes in biological systems and related physical phenomena.

## Supporting Information Available

Circular dichroism measurements of tripeptide solutions (Figure S1); representative XPS of LLL-tripeptide self-assembled monolayer (Figure S2); voltammetry of LLL-tripeptide with the C6OH diluent SAM and 11-MUA tripeptide assemblies before and after immobilization of Cyt c (Figure S3); surface coverage of tripeptide self-assembled monolayers (Table S1); experimental data for Figure 3 (Table S2); experimental data for Figure 3 (Table S3); experimental data of LLL-tripeptide assemblies in Figure 4 (Table S4); experimental data of DDD-tripeptide assemblies in Figure 4 (Table S5); and python script for data analysis (Supporting Note 1) (PDF)
