# Supplementary material for: Examining the Effects of Homochirality for Electron Transfer in Protein Assemblies
Source: J Phys Chem B. 2023 Jul 18;127(29):6462–9. doi: 10.1021/acs.jpcb.3c02913 (PMC10388353; doi:10.1021/acs.jpcb.3c02913)
Supplement: Supplementary file 1 — jp3c02913_si_001.pdf [file jp3c02913_si_001.pdf]

Supporting information for:

Examining the Effects of Homochirality for Electron Transfer in Protein Assemblies

*Jimeng Wei, Brian P. Bloom, Wiley A. Dunlap-Shohl, Caleb B. Clever, José E. Rivas, and David H. Waldeck**

Department of Chemistry, University of Pittsburgh, Pittsburgh, PA, USA

Email: dave@pitt.edu Phone: 412-624-8430

This PDF includes:

Figure S1 – Circular dichroism measurements of tripeptide solutions

Figure S2 – Representative XPS of LLL-tripeptide self-assembled monolayer

Figure S3 – Voltammetry of LLL-tripeptide with C6OH diluent SAM, and 11-MUA tripeptide, assemblies before and after immobilization of Cyt *c*

Table S1 – Surface coverage of tripeptide self-assembled monolayers

Table S2 – Experimental data from Figure 4A – pure tripeptide SAM assemblies

Table S3 – Experimental data from Figure 3B – diluted SAM assemblies

Table S4 – Experimental data of LLL-tripeptide assemblies in Figure 4

Table S5 – Experimental data of DDD-tripeptide assemblies in Figure 4

Supplementary Note 1 – Python script for data analysis

Figure S1 shows CD spectra for LLL-tripeptide (blue), DDD-tripeptides (red), an LDL-tripeptide solutions in the near UV region. As expected, the spectra show mirror image symmetry for the LLL-tripeptides and DDD-tripeptides.

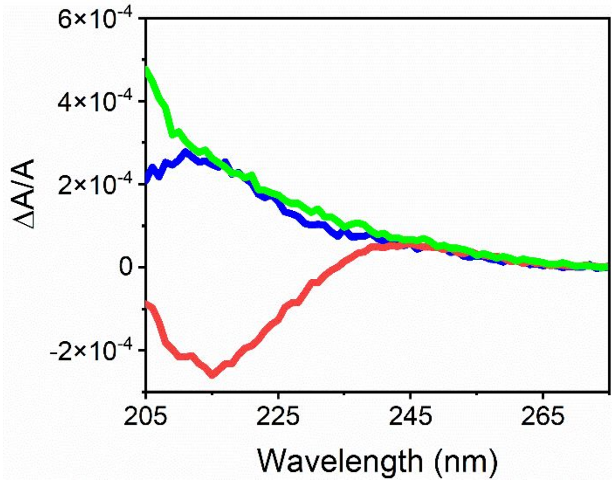

Figure S1. Circular dichroism spectra of 250μM LLL-tripeptide (blue), DDD-tripeptide (red), and LDL-tripeptide (green) solutions in ethanol.

To characterize the tripeptide SAM assemblies and ascertain the surface coverages, X-ray photoelectron spectroscopy (XPS) was employed. Figure S2 shows a representative S2p spectrum of an LLL-tripeptide SAM. The spectrum is fit to a doublet (blue), using the Avantage software package,[1]and the $S2p_{3/2}$ peak centered at ~162.0 eV is consistent with the formation of an Au-S bond. $S2p_{3/2}$ peaks at ~163.5 eV and 168.5 eV, corresponding to S-S / S-H and S-O bonds,[2] are not present in the spectra and indicate that rinsing removes any physisorbed peptides and that the thiol group is not oxidized. The surface coverage of the peptide SAM was quantified using previously established protocols.[3] Briefly, the atomic percent ratio of the Au to S peaks for the peptide SAM was compared to a reference system with a known surface coverage; 1-dodecanethiol (4.62 x 10^{14} molecules/cm^2).[4] The surface coverage was determined from the average of multiple points across three separately prepared samples; see Table S1. While this study does not account for the change in photoelectron escape depth between a tripeptide and an alkanethiol, these data indicate the formation of a dense SAM and that the coverages for LLL- and DDD-tripeptide are equal, to within instrumental error (see Table S1).

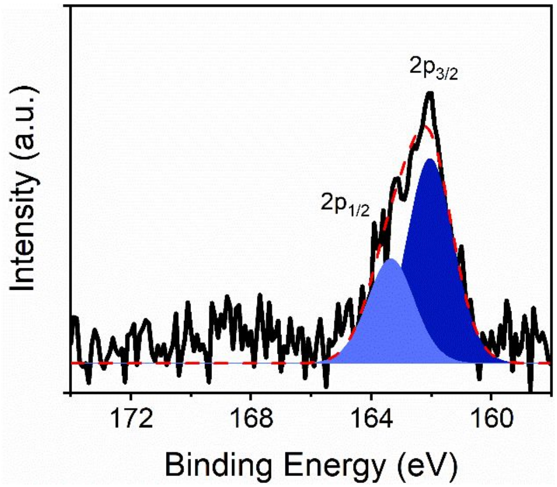

Figure S2. XPS S2p spectra of an LLL-tripeptide self-assembled monolayer. The shaded blue regions correspond to an $S2p_{3/2}$ and $S2p_{1/2}$ doublet fitting to the data and the red dashed line is an envelope to the fitting.

Table S1. Surface coverage of LLL-, DDD-, and LDL-tripeptide SAMs on gold determined by XPS.

	Coverage (x10^{14} molecules/cm^2)
LLL-tripeptide	4.82 ± 0.53
DDD-tripeptide	4.93 ± 0.76
LDL-tripeptide	4.16 ± 0.32

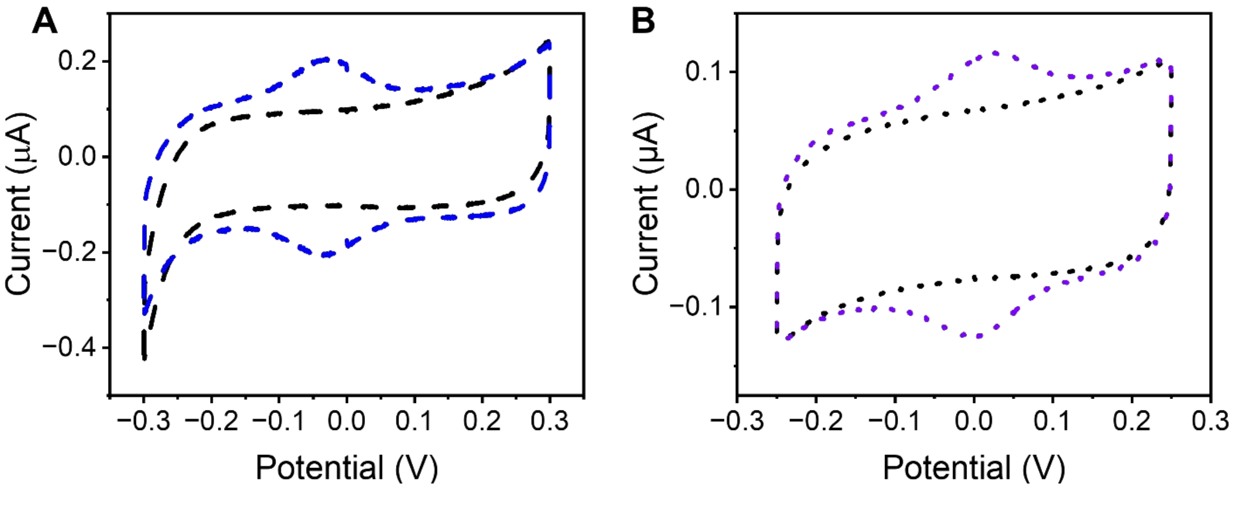

Figure S3. (A) Cyclic voltammogram of an LLL-tripeptide with C6OH diluent SAM before (black, dash) and after (blue, dash) immobilization of Cyt *c*. (B) Cyclic voltammogram of an 11-MUA SAM before (black, dot) and after (purple, dot) immobilization of Cyt *c*.

Table S2. KCl concentrations, asymmetry factors, and number of trials obtained for Cyt *c* immobilized on pure tripeptide SAMs in Figure 4. Uncertainty in the polarization is represented by the standard deviations taken among different trials.

SAM composition	KCl concentration (mM)	Average asymmetry factor	No. of trials
LLL-tripeptide	10	-0.26 ± 0.07	6
LLL-tripeptide	20	-0.21 ± 0.11	6
LLL-tripeptide	30	-0.10 ± 0.04	10
LLL-tripeptide	40	-0.11 ± 0.08	5
DDD-tripeptide	10	0.50 ± 0.14	6
DDD-tripeptide	20	0.32 ± 0.09	6
DDD-tripeptide	30	0.35 ± 0.06	12
DDD-tripeptide	40	0.48 ± 0.10	6
LDL-tripeptide	10	0.03 ± 0.02	5
LDL-tripeptide	20	0.04 ± 0.03	5
LDL-tripeptide	30	0.01 ± 0.01	11
LDL-tripeptide	40	-0.01 ± 0.04	5
11-MUA	10	0.02 ± 0.01	6
11-MUA	20	0 ± 0	6
11-MUA	30	0 ± 0	8
11-MUA	40	0.10 ± 0.05	6

Table S3. KCl concentrations, asymmetry factors, and number of trials obtained for Cyt *c* immobilized on tripeptide mixed with C6OH SAMs in Figure 3.

SAM composition	KCl concentration (mM)	Average asymmetry factor	No. of trials
LLL-tripeptide	10	-0.15 ± 0.02	6
LLL-tripeptide	20	-0.15 ± 0.03	6
LLL-tripeptide	30	-0.15 ± 0.03	4
LLL-tripeptide	40	-0.17 ± 0.02	6
DDD-tripeptide	10	0.15 ± 0.02	6
DDD-tripeptide	20	0.16 ± 0.03	6
DDD-tripeptide	30	0.13 ± 0.01	6
DDD-tripeptide	40	0.13 ± 0.02	6
LDL-tripeptide	10	0.01 ± 0.01	4
LDL-tripeptide	20	0 ± 0	6

LDL-tripeptide	30	0.03 ± 0.02	6
LDL-tripeptide	40	0 ± 0	6
11-MUA	10	0 ± 0	4
11-MUA	20	0.02 ± 0.04	4
11-MUA	30	0 ± 0	4
11-MUA	40	0.01 ± 0.01	4

Table S4. Value of KCl concentrations, <k^0>, and absolute asymmetry factors for LLL-tripeptide data in Figure 4.

KCl concentration (mM)	<k^0> (s^{-1})	$\lvert A \rvert$
30	163	0.154
30	135	0.074
30	185	0.270
30	158	0.222
30	208	0.120
30	150	0.133
10	275	0.327
10	210	0.571
20	305	0.295
30	305	0.230
30	265	0.038
40	245	0.122
10	225	0.133
10	210	0.154
20	190	0.211
20	160	0.667
40	170	0.353
10	215	0.140
10	265	0.264
20	275	0.182
20	200	0.100
30	310	0.006
30	215	0.140
40	270	0.148
40	260	0.008

Table S5. Value of KCl concentrations, <k^0>, and absolute asymmetry factors for DDD-tripeptide data in Figure 4.

KCl concentration (mM)	<k^0> (s^{-1})	$\lvert A \rvert$

30	32	0.222
30	33	0.769
30	35	0.286
30	19	0.378
30	36	0.254
30	18	0.286
10	63	0.400
10	48	0.316
20	45	0.222
20	35	0.286
30	38	0.400
40	38	0.400
10	36	0.535
10	10	1.158
20	27	0.642
20	4	0.000
30	19	0.270
30	13	0.400
40	14	0.222
40	12	0.261
10	90	0.222
10	50	0.400
20	85	0.353
20	50	0.400
30	80	0.500
30	40	0.500
40	70	0.571
40	35	0.857

Supplementary Note 1

Python programming for peak potential extraction

In many cases, fitting of peaks in cyclic voltammograms is complicated by the existence of a background due to double layer charging. The background can usually be satisfactorily approximated by a linear baseline whose slope and intercept are determined by visual inspection, but it is not always evident where the points on the curve should be selected to determine these parameters (particularly when the voltammogram's peaks are weak). This method may thus introduce significant variation into the calculation of the position and width of the peaks, as well as sensitivity to the subjective choice of the appropriate range over which to perform the baseline subtraction and fit the peak. To characterize this variation and extract the most accurate possible peak parameters, we have written a Python program to perform the peak fitting algorithm for an ensemble of reasonable choices of data used to estimate the double layer baseline, represented by bands of voltages on either side of each peak over which the component of the current originating from the Cyt *c* electron transfer process is plausibly negligible. All possible pairs of "start" and "end" voltages in these bands are used to determine a baseline, and the peak parameters are subsequently

determined by fitting a Voigt lineshape to the data remaining after subtracting this baseline. While this method cannot entirely remove the subjectivity in baseline estimation since the start and end bands must still be chosen at the researcher's discretion, providing a distribution of estimates for the peak position and FWHM can at least alleviate the bias to some degree by taking the means of these distributions as the result of an ensemble of reasonable guesses. Crucially, the standard deviations of the parameter distributions also provide estimates of how sensitive the fits are to these subjective choices, allowing us to characterize our confidence in the parameter values. The source code for this process (developed in the Jupyter Notebook environment) is reproduced below.

```
# Preamble: import useful modules and define functions that will be used later

import numpy as np
import pandas as pd
import scipy as sp
from scipy.optimize import curve_fit
from sklearn.metrics import r2_score
import matplotlib.pyplot as plt

# Voigt function for fitting - augment the standard SciPy function with shift and scale parameters

def Voigt_shifted(x, mu, c, sigma, gamma):
    # mu: peak center
    # c: scale factor
    # sigma: std. dev. of the Gaussian component
    # gamma: half-width half maximum of the Cauchy-Lorentz component

    s = x - mu
    profile = c*scipy.special.voigt_profile(s, sigma, gamma)
    return profile

import os
import re

# Define the folder path where the text files are located
folder_path = " C:/Users/simon/Desktop/Fitting data/Set 1/"

# Loop through each file in the folder
for filename in os.listdir(folder_path):
    # Check if the file is a text file
    if filename.endswith(".txt"):
        # Read the file
        with open(os.path.join(folder_path, filename), "r") as f:
            lines = f.readlines()
        # Find the line number where the data starts
        start_line = None
        for i, line in enumerate(lines):
            if re.search(r'Potential/V, Current/A', line):
                start_line = i
                break
        # If the data start line was found, overwrite the file with the updated contents
        if start_line is not None:
            with open(os.path.join(folder_path, filename), "w") as f:
                f.writelines(lines[start_line:])

# Setup: load data and visualize; use this cell to tweak the start and end bands
# before running the processing algorithm in the next cell

# construct the path to the CSV containing names of files to analyze and the bands corresponding
# to plausible start and end voltages for the anodic and cathodic peaks and import these data
# into a Pandas DataFrame - note that each CSV corresponding to a single experiment, conducted
# at varying scan rates

CV_path = 'C:/Users/simon/Desktop/Fitting data/'
subfolder = 'Set 1/'
key_CSV = 'Voltage Ranges subtracted.csv'
filedata = pd.read_csv(CV_path + subfolder + key_CSV)
files = filedata['Filename']

# if set to "True", this statement can be used to confirm that the start and end bands of the
# fitting ranges are appropriately set, at the expense of memory consumption

plot_rawdata = True
```

```
# best to leave this "False" - will plot every single peak fit and consume very large amounts of
# memory if not, but can also be a potentially useful diagnostic

plot_peak_data = False

# initialize list that will keep track of peak positions

scan_data = []

# account for different formatting of data files from different instruments -
# the main concern is how many lines in each data file are header and need to be
# skipped over

instrument = '1'

if instrument == '1':
    rows_to_skip = 20
elif instrument == '2':
    rows_to_skip = 39

# loop over rows in the CSV (again, corresponding to different scan rates)

for kk, filename in enumerate(files):

    # extract the scan rate from the filename itself; exact details of how the string
    # should be parsed will vary according to how the files are named.

    file = CV_path + subfolder + filename
    scanrate = float(filename.split('mVs')[0].split('Cyt c ')[1])

    # print status update - analysis can be slow

    print('\n---------------------------------------------------')
    print('Status Update: Analyzing ' + filename + '\n')

    # 1. Read in the raw data and plot them, saving figures for visualization

    data = pd.read_csv(file,skiprows=rows_to_skip,names=['Potential (V)','Current (A)'])
    voltage = data['Potential (V)'].values
    current = data['Current (A)'].values

    # 2. Define the fit start and end ranges for both sweep directions

    Vstart_a_anodic = filedata['V_start_a_anodic'][kk]
    Vstart_b_anodic = filedata['V_start_b_anodic'][kk]
    Vend_a_anodic = filedata['V_end_a_anodic'][kk]
    Vend_b_anodic = filedata['V_end_b_anodic'][kk]

    Vstart_a_cathodic = filedata['V_start_a_cathodic'][kk]
    Vstart_b_cathodic = filedata['V_start_b_cathodic'][kk]
    Vend_a_cathodic = filedata['V_end_a_cathodic'][kk]
    Vend_b_cathodic = filedata['V_end_b_cathodic'][kk]

    # plot the raw data if desired (set using the boolean variable "plot_rawdata" defined above)

    if plot_rawdata:
        plt.figure()
        plt.plot(voltage,current)
        plt.xlabel('Potential (V)')
        plt.ylabel('Current (A)')

        # plot the fitting ranges as colored bars for easy visualization

        I_min = np.min(current)
        I_max = np.max(current)

        plt.fill([Vstart_a_anodic,Vstart_b_anodic,Vstart_b_anodic,Vstart_a_anodic,Vstart_a_anodic],
                 [0,0,I_max,I_max,0],
                 color='xkcd:burgundy',alpha=0.5,
                 label='Anodic - Start'
                )

        plt.fill([Vend_a_anodic,Vend_b_anodic,Vend_b_anodic,Vend_a_anodic,Vend_a_anodic],
                 [0,0,I_max,I_max,0],
                 color='xkcd:scarlet',alpha=0.5,
                 label='Anodic - End'
                )

        plt.fill([Vstart_b_cathodic,Vstart_a_cathodic,Vstart_a_cathodic,Vstart_b_cathodic,Vstart_b_cathodic],
```

```
            [I_min,I_min,0,0,I_min],
            color='xkcd:cobalt blue',alpha=0.5,
            label='Cathodic - Start'
            )

    plt.fill([Vend_b_cathodic,Vend_a_cathodic,Vend_a_cathodic,Vend_b_cathodic,Vend_b_cathodic],
            [I_min,I_min,0,0,I_min],
            color='xkcd:cerulean',alpha=0.5,
            label='Cathodic - End'
            )

    plt.legend(frameon=False, fontsize = 16)

    # save the plot to the same directory as the one containing the raw data

    plt.savefig(file.split('.txt')[0] + '.png', format='png', bbox_inches='tight', pad_inches = 1)

# Processing: divide each full CV trace into separate sweeps, subtract the background, fit the peak to a
# Voigt function, and collect all the fit parameters for each and save to summary CSVs

# 3. Extract the different sweep segments from the full trace

scan_no = 0 # index tracking which scan we're on
rising_V = True # is the voltage going up or down; by default, rising first
switch_idxs = [0] # keep track of where the voltage shifts direction
scan_dirs = [True] # scan direction - True if voltage rising during scan (anodic sweep),
                  #                   False if falling (cathodic sweep)

# determine endpoints of each voltage scan and their directions

for ii in range(len(voltage) - 1):
    if rising_V and (voltage[ii+1] < voltage[ii]):
        rising_V = False
        scan_dirs.append(rising_V)
        switch_idxs.append(ii)
        # print('Switch from rising to falling potential')
    elif (not rising_V) and (voltage[ii+1] > voltage[ii]):
        rising_V = True
        scan_dirs.append(rising_V)
        switch_idxs.append(ii)
        # print('Switch from falling to rising potential')

    # update voltage sweep direction

    rising_V = True if voltage[ii+1] > voltage[ii] else False

switch_idxs.append(-1) # add the endpoint of the final sweep
Nscans = len(switch_idxs) – 1 # calculate how many scans are in each file

# 4. Prepare to loop over each scan

# keep track of which sweep we're on

anodic_count = 1
cathodic_count = 1

# initialize lists of parameters for each fit

anodic_data = []
cathodic_data = []

# set up figures to look at processed peaks and fits if desired (set using boolean variable
# "plot_peak_data" defined above)

if plot_peak_data:
    a_fig, a_ax = plt.subplots()
    c_fig, c_ax = plt.subplots()

# 5. Loop over anodic and cathodic sweeps - note that the first sweep in each direction is
# neglected in order to reject the effects of initial transient behavior - i.e., the loop starts at
# index value 2 instead of 0

for jj in range(2,Nscans,1):

    # get the data corresponding to a single scan

    full_range_V = voltage[switch_idxs[jj]:switch_idxs[jj+1]]
    full_range_I = current[switch_idxs[jj]:switch_idxs[jj+1]]

    # if anodic sweep:
```

```
if scan_dirs[jj]:

    # status update

    print('Status Update: Fitting Anodic Sweep #',anodic_count,'...')

    # define the explicit ranges of voltages to start and end at

    start_band = [V for V in full_range_V if (V >= Vstart_a_anodic and V <= Vstart_b_anodic)]
    end_band = [V for V in full_range_V if (V >= Vend_a_anodic and V <= Vend_b_anodic)]

    # iterate over each possible start and endpoint for the fit

    for V_start in start_band:

        for V_end in end_band:

            # identify the corresponding indices

            start_idx = np.argmin(np.abs(full_range_V - V_start))
            end_idx = np.argmin(np.abs(full_range_V - V_end))

            # specify the fitting range for each specific combination of start and end

            V_range_red = full_range_V[start_idx:end_idx+1]
            I_range_red = full_range_I[start_idx:end_idx+1]

            # get current at the endpoints

            I_start = I_range_red[0]
            I_end = I_range_red[-1]

            # fit a linear background and subtract it

            I_bkg = I_start + ((I_end - I_start)/(V_end - V_start))*(V_range_red - V_start)
            I_peak_full = I_range_red - I_bkg

                        # plot the scans if desired

            if plot_peak_data:

                a_ax.plot(V_range_red,I_peak_full)

            # get raw peak parameters - too noisy for reasonable implementation
            # but provide good initial guesses for fits

            V_peak = V_range_red[np.argmax(I_peak_full)] # voltage at the peak
            I_peak = I_peak_full[np.argmax(I_peak_full)]

            try:
                # fit a Voigt function to the peak

                popt, pcov = scipy.optimize.curve_fit(Voigt_shifted,
                                                      V_range_red,
                                                      I_peak_full,
                                                      p0=(V_peak,I_peak,0.05,0.02)
                                                     )
                peak_R2 = r2_score(I_peak_full,Voigt_shifted(V_range_red,*popt))

                # test GoF by plotting

                if plot_peak_data:
                    a_ax.plot(V_range_red,Voigt_shifted(V_range_red,*popt),'--')
                    a_ax.set_xlabel('Potential (V)')
                    a_ax.set_ylabel('Current (A)')
                    a_ax.set_title('$R^2$ = ' + str(np.round(peak_R2,4)))

                # extract peak parameters

                peak_center = popt[0]
                peak_scale = popt[1]
                peak_sigma = popt[2]
                peak_gamma = popt[3]

                # approximation to Voigt function FWHM, accurate to <0.02%
                peak_FWHM = 0.5346*2*peak_gamma + np.sqrt(0.2166*(2*peak_gamma)**2 +\
                                          (2*peak_sigma*np.sqrt(2*np.log(2)))**2)

                # collect all the fit parameters and add them to the running list
```

```
                anodic_params = [anodic_count,
                                 V_start,
                                 V_end,
                                 peak_center,
                                 peak_scale,
                                 peak_sigma,
                                 peak_gamma,\
                                 peak_FWHM,
                                 peak_R2
                                ]
                anodic_data.append(anodic_params)

            # sometimes the fit fails to converge; if so, pass by that particular combination
            # of start and end voltages

            except:
                pass

    # after iterating through the full start and stop bands, update the sweep counter
    anodic_count += 1

# if cathodic sweep:
else:

    # status update

    print('Status Update: Fitting Cathodic Sweep #',cathodic_count,'...')

    # define the explicit ranges of voltages to start and end at

    start_band = [V for V in full_range_V if (V <= Vstart_a_cathodic and V >= Vstart_b_cathodic)]
    end_band = [V for V in full_range_V if (V <= Vend_a_cathodic and V >= Vend_b_cathodic)]

    # iterate over each possible start and endpoint for the fit

    for V_start in start_band:
        for V_end in end_band:

            # identify the corresponding indices

            end_idx = np.argmin(np.abs(full_range_V - V_start))
            start_idx = np.argmin(np.abs(full_range_V - V_end))

            # specify the fitting range

            V_range_red = full_range_V[start_idx:end_idx+1]
            I_range_red = full_range_I[start_idx:end_idx+1]

            # get current at the endpoints

            I_start = I_range_red[-1]
            I_end = I_range_red[0]

            # fit a linear background and subtract it

            I_bkg = I_start + ((I_end - I_start)/(V_end - V_start))*(V_range_red - V_start)
            I_peak_full = I_bkg - I_range_red

                                # plot the scans if desired

            if plot_peak_data:

                c_ax.plot(V_range_red,I_peak_full)

            # get raw peak parameters - too noisy for reasonable implementation
            # but provide good initial guesses for fits

            V_peak = V_range_red[np.argmax(I_peak_full)] # voltage at the peak
            I_peak = I_peak_full[np.argmax(I_peak_full)]

            try:
                # fit a Voigt function to the peak

                popt, pcov = scipy.optimize.curve_fit(Voigt_shifted,
                                                      V_range_red,
                                                      I_peak_full,
                                                      p0=(V_peak,I_peak,0.05,0.02)
                                                     )
                peak_R2 = r2_score(I_peak_full,Voigt_shifted(V_range_red,*popt))
```

```
                    # test GoF by plotting

            if plot_peak_data:
                c_ax.plot(V_range_red,Voigt_shifted(V_range_red,*popt),'--')
                c_ax.set_xlabel('Potential (V)')
                c_ax.set_ylabel('Current (A)')
                c_ax.set_title('$R^2$ = ' + str(np.round(peak_R2,4)))

            # extract peak parameters

            peak_center = popt[0]
            peak_scale = popt[1]
            peak_sigma = popt[2]
            peak_gamma = popt[3]

            # approximation to Voigt function FWHM accurate to <0.02%

            peak_FWHM = 0.5346*2*peak_gamma + np.sqrt(0.2166*(2*peak_gamma)**2 +\
                                  (2*peak_sigma*np.sqrt(2*np.log(2)))**2)

            # collect all the fit parameters and add them to the running list

            cathodic_params = [cathodic_count,
                         V_start,
                         V_end,
                         peak_center,
                         peak_scale,
                         peak_sigma,
                         peak_gamma,
                         peak_FWHM,
                         peak_R2
                         ]
            cathodic_data.append(cathodic_params)

        # sometimes the fit fails to converge; if so, pass by that particular combination
        # of start and end voltages

        except:
            pass

    # after iterating through the full start and stop bands, update the sweep counter

    cathodic_count += 1

# collect all the peak fitting data into a DataFrame

anodic_DF = pd.DataFrame(data=anodic_data,
             columns=['Sweep #','Start Voltage (V)','End Voltage (V)',
                 'Peak Center (V)', 'Peak Scale (A)',
                 'Peak Sigma (V)', 'Peak Gamma (V)', 'Peak FWHM (V)',
                 'Fit R2'
                 ]
            )

# collect all the peak fitting data into a DataFrame

cathodic_DF = pd.DataFrame(data=cathodic_data,
              columns=['Sweep #','Start Voltage (V)','End Voltage (V)',
                  'Peak Center (V)', 'Peak Scale (A)',
                  'Peak Sigma (V)', 'Peak Gamma (V)', 'Peak FWHM (V)',
                  'Fit R2'
                  ]
             )

# save the data as a CSV in the same folder

anodic_DF.to_csv(file.split('.txt')[0] + '_anodic_sweep_fit_params.csv')
cathodic_DF.to_csv(file.split('.txt')[0] + '_cathodic_sweep_fit_params.csv')

# get basic statistics on the peak centers

anodic_peakcenter_mean = np.mean(anodic_DF['Peak Center (V)'])
anodic_peakcenter_std = np.std(anodic_DF['Peak Center (V)'])
anodic_fwhm_mean = np.mean(anodic_DF['Peak FWHM (V)'])
anodic_fwhm_std = np.std(anodic_DF['Peak FWHM (V)'])

cathodic_peakcenter_mean = np.mean(cathodic_DF['Peak Center (V)'])
cathodic_peakcenter_std = np.std(cathodic_DF['Peak Center (V)'])
cathodic_fwhm_mean = np.mean(cathodic_DF['Peak FWHM (V)'])
cathodic_fwhm_std = np.std(cathodic_DF['Peak FWHM (V)'])
```

```
    # finally, update the list tracking peak centers/standard deviations along with scan rates

    scan_data.append([scanrate,
                      anodic_peakcenter_mean,
                      anodic_peakcenter_std,
                      cathodic_peakcenter_mean,
                      cathodic_peakcenter_std,
                      anodic_fwhm_mean,
                      anodic_fwhm_std,
                      cathodic_fwhm_mean,
                      cathodic_fwhm_std,
                     ])

# turn the scan rate list into a DataFrame with the appropriate headings

grand_DF = pd.DataFrame(data=scan_data,
                        columns=['Scan Rate (mV/s)',
                                 'Anodic Peak Mean (V)',
                                 'Anodic Peak SD (V)',
                                 'Cathodic Peak Mean (V)',
                                 'Cathodic Peak SD (V)',
                                 'Anodic FWHM Mean (V)',
                                 'Anodic FWHM SD (V)',
                                 'Cathodic FWHM Mean (V)',
                                 'Cathodic FWHM SD (V)'
                                ]
                        )

# calculate the formal reduction potential as the mean of the peak positions

grand_DF['Formal Potential (V)'] = (grand_DF['Anodic Peak Mean (V)'] + grand_DF['Cathodic Peak Mean (V)'])/2

# make trumpet plot using the differences between peak positions and the formal potential

fig, ax = plt.subplots()
ax.errorbar(grand_DF['Scan Rate (mV/s)'],
            grand_DF['Anodic Peak Mean (V)']-grand_DF['Formal Potential (V)'],
            yerr=grand_DF['Anodic Peak SD (V)'],
            capsize=5,marker='o',color='k',linestyle=' ',label='Anodic Scans')
ax.errorbar(grand_DF['Scan Rate (mV/s)'],
            grand_DF['Cathodic Peak Mean (V)']-grand_DF['Formal Potential (V)'],
            yerr=grand_DF['Cathodic Peak SD (V)'],
            capsize=5,marker='o',color='r',linestyle=' ',label='Cathodic Scans')
ax.set_xscale('log')
ax.set_xlabel('Scan Rate (mV/s)')
ax.set_ylabel('$E_\mathrm{peak} - E^\circ\' $ (V)')
ax.set_title('Symmetrized Trumpet Plot')
ax.legend(frameon=False)

# make trumpet plot using the differences between peak positions and the formal potential

fig, ax = plt.subplots()
ax.errorbar(grand_DF['Scan Rate (mV/s)'],
            grand_DF['Anodic Peak Mean (V)'],
            yerr=grand_DF['Anodic Peak SD (V)'],
            capsize=5,marker='o',color='k',linestyle=' ',label='Anodic Scans')
ax.errorbar(grand_DF['Scan Rate (mV/s)'],
            grand_DF['Cathodic Peak Mean (V)'],
            yerr=grand_DF['Cathodic Peak SD (V)'],
            capsize=5,marker='o',color='r',linestyle=' ',label='Cathodic Scans')
ax.set_xscale('log')
ax.set_xlabel('Scan Rate (mV/s)')
ax.set_ylabel('$E_\mathrm{peak}$ (V)')
ax.set_title('Asymmetric Trumpet Plot')
ax.legend(frameon=False)

# visualize the tabulated results

grand_DF
```

REFERENCES

1 *Thermo Avantage*, v5.977 Build 06436; Thermo Fisher Scientific: 2017.

2 Ulman, A.; Ioffe, M.; Patolsky, F.; Haas, E.; Reuvenov, D., Highly active engineered-enzyme oriented monolayers: formation, characterization and sensing applications. *J. Nanobiotechnology* **2011,** *9* (1), 26.

3 Yawitz, T. M.; Patterson, K. S.; Onkst, B. X.; Youmbi, F.; Clark, R. A., Cytochrome *c* electrochemistry on peptide self-assembled monolayers. *J. Electroanal. Chem.* **2018,** *828*, 59-62.

4 Marvin, C. W.; Grimm, H. M.; Miller, N. C.; Horne, W. S.; Hutchison, G. R., Interplay among Sequence, Folding Propensity, and Bio-Piezoelectric Response in Short Peptides and Peptoids. *J. Phys. Chem. B* **2017,** *121* (44), 10269-10275.
